# Home-Based Virtual Reality-Augmented Training Improves Lower Limb Muscle Strength, Balance, and Functional Mobility following Chronic Incomplete Spinal Cord Injury

**DOI:** 10.3389/fneur.2017.00635

**Published:** 2017-11-28

**Authors:** Michael Villiger, Jasmin Liviero, Lea Awai, Rahel Stoop, Pawel Pyk, Ron Clijsen, Armin Curt, Kynan Eng, Marc Bolliger

**Affiliations:** ^1^Department of Business Economics, Health and Social Care, University of Applied Sciences and Arts of Southern Switzerland, Landquart, Manno, Switzerland; ^2^THIM University of Applied Sciences, Landquart, Switzerland; ^3^Institute of Human Movement Science and Sport, ETH Zurich, Zurich, Switzerland; ^4^Institute of Neurology, University College London, London, United Kingdom; ^5^Spinal Cord Injury Center, University Hospital Balgrist, Zurich, Switzerland; ^6^Institute of Neuroinformatics, University of Zurich and ETH Zurich, Zurich, Switzerland

**Keywords:** neurological rehabilitation, virtual reality therapy, spinal cord injuries, motor function, lower extremity

## Abstract

Key factors positively influencing rehabilitation and functional recovery after spinal cord injury (SCI) include training variety, intensive movement repetition, and motivating training tasks. Systems supporting these aspects may provide profound gains in rehabilitation, independent of the subject’s treatment location. In the present study, we test the hypotheses that virtual reality (VR)-augmented training at home (i.e., unsupervised) is feasible with subjects with an incomplete SCI (iSCI) and that it improves motor functions such as lower limb muscle strength, balance, and functional mobility. In the study, 12 chronic iSCI subjects used a home-based, mobile version of a lower limb VR training system. The system included motivating training scenarios and combined action observation and execution. Virtual representations of the legs and feet were controlled via movement sensors. The subjects performed home-based training over 4 weeks, with 16–20 sessions of 30–45 min each. The outcome measures assessed were the Lower Extremity Motor Score (LEMS), Berg Balance Scale (BBS), Timed Up and Go (TUG), Spinal Cord Independence Measure mobility, Walking Index for Spinal Cord Injury II, and 10 m and 6 min walking tests. Two pre-treatment assessment time points were chosen for outcome stability: 4 weeks before treatment and immediately before treatment. At post-assessment (i.e., immediately after treatment), high motivation and positive changes were reported by the subjects (adapted Patients’ Global Impression of Change). Significant improvements were shown in lower limb muscle strength (LEMS, *P* = 0.008), balance (BBS, *P* = 0.008), and functional mobility (TUG, *P* = 0.007). At follow-up assessment (i.e., 2–3 months after treatment), functional mobility (TUG) remained significantly improved (*P* = 0.005) in contrast to the other outcome measures. In summary, unsupervised exercises at home with the VR training system led to beneficial functional training effects in subjects with chronic iSCI, suggesting that it may be useful as a neurorehabilitation tool.

**Trial registration:**

Canton of Zurich ethics committee (EK-24/2009, PB_2016-00545), ClinicalTrials.gov: NCT02149186. Registered 24 April 2014.

## Introduction

An injured spinal cord leads to diminished functional recovery. The injured axons are limited in their spontaneous regeneration and compensatory fiber growth (1–3). From pre-clinical studies it is known that active rehabilitative training is crucial to promote and enhance functional recovery (4). Important characteristics of rehabilitation training such as task-specificity, task-variability or performance feedback have been identified (5, 6). Augmented exercise therapy has been shown to have favorable effects on rehabilitation outcomes in subjects with incomplete spinal cord injury (iSCI) and stroke (7, 8), and longer training duration is referred to higher walking function (dose–response context) (9, 10). Beside the amount of training, active participation is a key element for functional improvements (11). To increase active participation of subjects during therapy sessions virtual reality (VR) scenarios may have a beneficial effect on motivation (12, 13) and increase rehabilitation success (14, 15). VR is an innovative technology that describes a scenario generated by a computer (virtual environment) in which the users can interact. Furthermore, it is possible to provide biofeedback and multimodal sensory stimuli which can be interactively used (16).

Based on these findings and a previous study by our group (7), the overall goal of this study was to assess the feasibility and efficacy of using an interactive VR rehabilitation device providing intensive, repetitive but specific movement therapy in lower limb movement rehabilitation, in situations where constant one-to-one therapist supervision and coaching is not possible. In the previous study, it was shown that subjects with iSCI improved in lower limb muscle strength, balance during functional activities and walking capacities in a clinic-based setting (i.e., one-to-one therapist supervision and coaching) during a VR-augmented training (7).

Therefore, in this study, we hypothesized that unsupervised home-based VR-augmented neurorehabilitation training is feasible in subjects with an iSCI and that it would improve their motor functions (i.e., muscle strength, balance, and mobility).

## Materials and Methods

### Subjects

Outpatients with a chronic (time since injury >1 year) iSCI (age 41–74 years) from the University Hospital Balgrist (Zurich, Switzerland) were included in the study by a medical referral between 2013 and 2015, and the sample size was restricted by this time period (Table 1). Inclusion criteria were as follows: subjects with a chronic iSCI and a lesion level below C4, no assistive and supporting systems needed to sit in a chair, and American Spinal Injury Association Impairment Scale (AIS) C or D (i.e., sensorimotor incomplete) at time of inclusion (C = more than half of the key muscles below the neurological level have a muscle grade less than 3; D = at least half of the key muscles below neurological level have muscle grade ≥3) (17).

**Table 1:** Clinical characteristics of participants with incomplete spinal cord injury.

Participants	Etiology	AIS	Level of injury	Years since injury	LEMS	BBS	WISCI II
P1	T	D	C7	11	47	55	20
P2	ME	D	C5	6	44	47	19
P3	T	C	C5	12	na	na	13
P4	ME	D	T4	6	40	48	16
P5	ME	D	T9	7	29	11	13
P6	T	C	T12	2	18	24	12
P7	T	D	T9	17	47	55	20
P8	T	D	T12	14	32	24	12
P9	ME	D	C7	6	46	55	20
P10	T	D	C4	6	46	51	20
P11	T	D	L3	4	41	35	19
P12	T	D	C5	5	43	47	16

*T, trauma; ME, medical etiology; AIS, ASIA (American Spinal Injury Association) Impairment Scale (C, D, sensorimotor incomplete); LEMS, Lower Extremity Motor Score; BBS, Berg Balance Scale; WISCI II, Walking Index for Spinal Cord Injury II; na, not applicable*.

Exclusion criteria were as follows: head injuries leading to cognitive or visual impairment, spasticity limiting lower limb movements, medication influencing therapy, and psychiatric limitation, depressive symptoms (i.e., score >14, Beck Depression Inventory) (18) or other neurological disorders.

The purpose of the study was clear to all subjects, and written informed consent was acquired. The experimental protocol was in accordance with the Declaration of Helsinki and approved by the Canton of Zurich ethics committee (EK-24/2009, PB2016-00545).

### VR-Augmented Training at Home (Home-Based Therapy)

**Figure 1:**
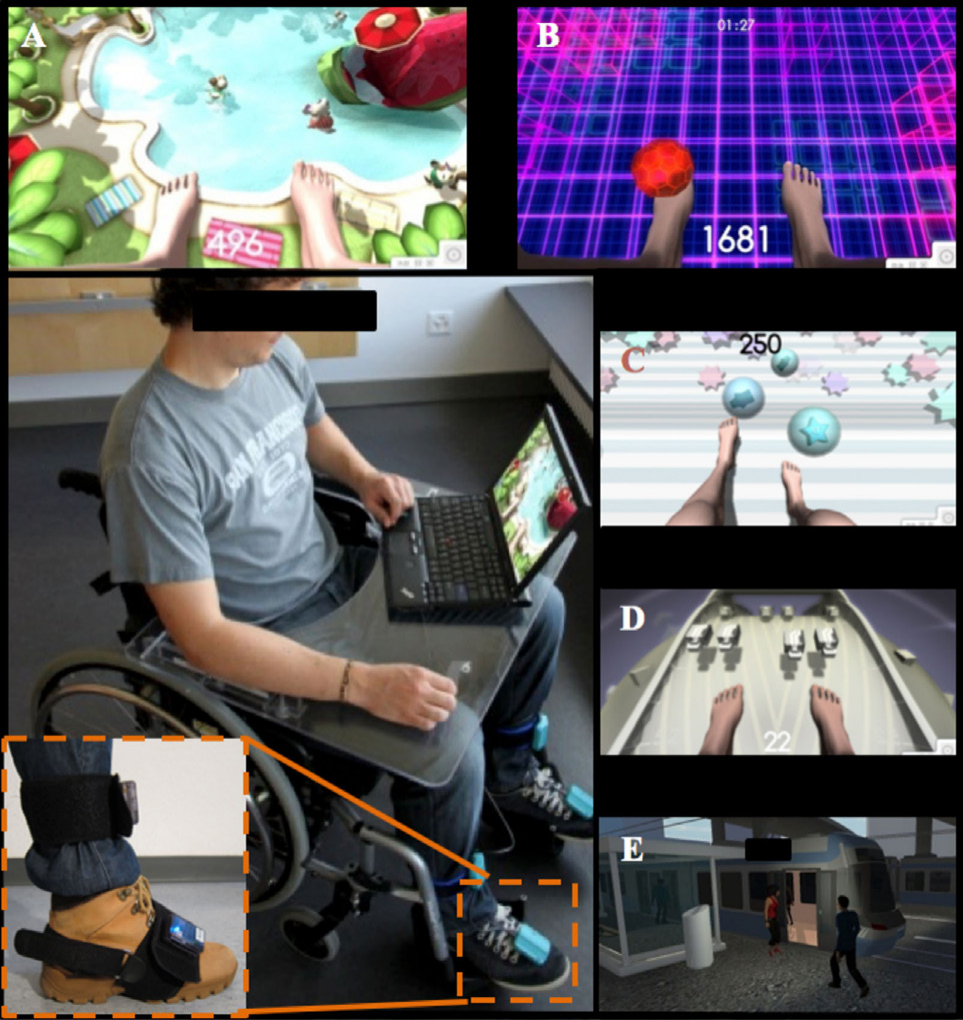
Overview of a virtual reality training setup and the different scenarios for training the various lower limb muscles and functions. Ankle dorsal flexion: Hamster Splash—launching hamsters into a swimming pool **(A)**; Footbag—juggling a ball **(B)**; and Get to the Game—walking from home via a tram station to a stadium **(E)**. Knee extension: Star Kick—kicking balls toward stars **(C)**. Leg ad-/abduction: planet drive—avoid touching oncoming cars **(D)**.

Home-based VR-augmented training was done with the mobile prototype of the YouKicker system (YouRehab AG, Schlieren, Switzerland) for the lower limbs, which is the successor of the stationary therapy system YouKicker described previously in a clinic-based study (standard one-on-one therapist-patient sessions at the University Hospital Balgrist) (7, 19). The home-based VR-augmented therapy system presented virtual representations of the feet and legs in a first-person perspective on a laptop computer and trained the lower limbs by combining action observation and execution (Figure 1). The system used four wireless 3 degree of freedom accelerometer sensor nodes that were attached bilaterally to the dorsum of the feet and the tibia, measuring inclination angles and transmitting movements to a laptop for processing and display on the screen. The foot sensor nodes were fastened on the foot with Velcro fixed to modified elastic rubber overshoes. The overshoes were worn over the patient’s own shoes. The tibia sensor nodes were fastened on the lower leg with Velcro and adjustable straps. Sensor data were sent via Bluetooth to a laptop computer using the Unity 3D game engine (Unity Technologies, San Francisco, CA, USA).

In collaboration with therapists, clinically relevant exercises for foot and leg while seated or in a standing position were generated. Five VR scenarios were provided on the training system to train foot and leg movements in sitting and standing positions. The system contained applications to train different isolated movements as follows.

Ankle dorsal flexion (three scenarios): *Footbag* (sitting/standing): the subject juggles a ball between the left and right foot. An ankle dorsal flexion (approx. 35/min per leg depending on patient ability) is performed preventing foot drag. The path of the ball is pre-set. *Hamster Splash* (*sitting*): hamsters rush to the subject’s feet. With a dorsal flexion of the ankle (approx. 15/min per leg) the subject launches each hamster into a swimming pool. Higher scores are achieved when faster ankle movement are performed. This also leads to more elaborate hamster movements (somersaults, swimming patterns). *Get to the Game*—*Activity of Daily Living* (sitting/standing): a virtual person (avatar), representing the subject, walks from home via a tram station to a stadium, as fast as possible. To make the avatar move, the subject has to alternately lift his/her feet. By increasing the range of motion of the ankle and by faster ankle movements, the subject can achieve a better score due to faster and bigger footsteps.

Knee extension (one scenario): *Star Kick* (sitting/standing): knee extensions are performed by the subjects (approx. 12/min per leg) hitting a ball toward presented stars with foot. For every kick, the subject scores points. With this exercise, the subject trains knee extension not using direct measurement of the knee angle.

Leg ad-/abduction (one scenario): *Planet Drive* (*sitting*): Cars are driving on a roadway toward virtual feet. The lower legs are tilted sideways (approx. 4/min per leg) to avoid touching the cars.

### Study Design

All subjects were trained at home on the VR tasks over a period of 4 weeks, with 16–20 sessions of 30–45 min each. The following information was collected at the beginning of the study: age, gender, height, weight, etiology, and level of lesion, AIS, time since injury, medication, pain presence, and intensity. In addition, the motivation was scored by the subjects on an 11-point Numeric Rating Scale (NRS) from 0 (i.e., worst) to 10 (i.e., best) after the completion of each training session.

The training system was set up by a therapist at the subject’s home. The subject completed the first training under the supervision of the therapist and on a weekly basis the therapist visited the subject to check the training and collect data. During a typical training session, each subject completed either one block (Footbag: 3 × 2 min, Planet Drive: 2 × 2.5 min, Star Kick: 2 × 2 min, Hamster Splash: 3 × 2 min) of training and a Get to the Game session (approx. 5 min), or twice the block without a Get to the Game session. The tasks were always presented in the same order during a session using the muscle groups alternately to prevent fatigue. Around 500 repetitions of ankle movements (Footbag, Hamster Splash, and Get to the Game) and 100 knee movements (Star Kick) with each leg are performed by a typical patient during a training session. When a subject came either to the same and/or higher number of repetitions in three successive sessions (i.e., increase in difficulty) or the number of repetitions was less in three successive sessions (i.e., decrease in difficulty) during the week the therapist changed the difficulty level of the task. A higher difficulty level resulted in higher speed or number (i.e., in more repetitions) of the specific target movements.

### Outcome Measures

All outcome measures were tested at the University Hospital Balgrist. For outcome stability for the subjects with iSCI before the intervention, two different assessment time points were chosen before treatment: 4 weeks before treatment program, i.e., *pre-baseline* assessments, and directly before treatment program, i.e., *baseline* assessments. After finishing of the training program, *post-assessments* were performed, and 2–3 months after treatment program, *follow-up* assessments were performed. The results at the four different assessment time points are presented in Table 2.

**Table 2:** Assessment time points and primary and secondary outcome measures.^a^

Assessment time points	Pre-baseline	Baseline	Treatment	Post^b^	Follow-up^b^
Weeks	−4	0	1–4	5	10–13
**Primary outcomes**					
LEMS (*n* = 11)	39.1 ± 7.5	39.5 ± 6.8	–	42.0 ± 7.6*	40.4 ± 8.6
BBS (*n* = 11)	41.6 ± 12.9	41.5 ± 12.7	–	43.3 ± 12.6*	42.6 ± 12.6
TUG (*n* = 10)	15.9 ± 7.9	15.4 ± 7.5	–	14.4 ± 6.8*	14.2 ± 6.8*
**Secondary outcomes**					
10MWT (*n* = 10)	1.09 ± 0.53	1.09 ± 0.51	–	1.13 ± 0.52	1.13 ± 0.53
6minWT (*n* = 10)	326.7 ± 176.7	324.5 ± 155.9	–	334.8 ± 157.4	337.9 ± 166.5
SCIM III mobility (*n* = 11)	31.2 ± 6.9	30.9 ± 7.2	–	32.6 ± 6.5	32.6 ± 7.1
WISCI II (*n* = 11)	17.0 ± 2.9	17.0 ± 2.9	–	17.6 ± 2.9	17.1 ± 3.1
PGIC (*n* = 11)	–	–	–	4.0 ± 1.3	–

*LEMS, Lower Extremity Motor Score (maximum points = 50); BBS, Berg Balance Scale (maximum points = 56); TUG, Timed Up and Go (s); 10MWTmax, 10 m walking test at fastest safely possible speed (m/s); 6minWT, 6 min walking test (m); SCIM III mobility, Spinal Cord Independence Measure III mobility (maximum points = 40); WISCI II, Walking Index for Spinal Cord Injury II (maximum points = 20); PGIC, Patients’ Global Impression of Change (adapted, maximum points = 7)*.

^a^Values are given as mean ± SEM and the significance level is set at *P ≤ 0.017.

*^b^Comparison to the averaged pre-baseline and baseline values using Wilcoxon signed-rank test*.

Outcome measures were included to test muscle strength, balance, and mobility, and for each field, the most conclusive outcome measure was chosen as primary outcome before testing: Lower Extremity Motor Score (LEMS) for lower limb muscle strength, Berg Balance Scale (BBS) for balance during functional activities, and the outcome Timed Up and Go (TUG), which covers functional mobility. This test correlates with performance in other mobility/walking outcomes, e.g., 10 m walking test (10MWT) and Walking Index for Spinal Cord Injury (WISCI) II, in subjects with iSCI (20). All other outcome measures were chosen as secondary outcome measures: 10MWT, 6 min walking test (6minWT), Spinal Cord Independence Measure (SCIM) III mobility, and WISCI II.

The minimal clinically important differences (MCIDs) are listed in the following section, if available for SCI. MCID is the minimal change of an assessment considering the clinical importance for the subject or clinician.

#### Primary Outcome Measures

##### Lower Limb Muscle Strength

From 0 (i.e., complete paralysis) to 50 (i.e., normal strength), which is measured by the LEMS (17). The MCID for the LEMS is 3.66 points (21).

##### Balance during Functional Activities

14 balance items, each from 0 (i.e., no balance) to 4 (i.e., good balance), are measured by the BBS (22). The MCID for the BBS is not established for SCI.

##### Functional Mobility—Transfer Ability

The TUG assesses various aspects of mobility in seconds (the subject has to stand up, walk 3 m forward, turn and walk back, and sit down) (20). The MCID for the TUG is not established for SCI.

#### Secondary Outcome Measures

##### Walking Speed/Distance

With the 10MWT, the time in seconds is measured by walking 10 m and with the 6minWT, and the distance in meter is measured by walking 6 min (23, 24). The MCID values calculated as the speed based on 10MWT and 6minWT are >0.05 and 0.1 m/s, respectively (25, 26).

##### Mobility

The use of assistive devices is assessed by the WISCI II from 0 (i.e., unable to walk) to 20 (i.e., able to walk without assistive devices) (27, 28). A clinically important difference is when a change of one WISCI level occurs (29). The SCIM mobility measures the transfer and indoor/outdoor mobility from 0 (i.e., no mobility) to 40 (i.e., normal mobility). The MCID for the SCIM is not established for SCI.

##### Self-Reported Change

The Patients’ Global Impression of Change (PGIC) (30) was adapted for motor function in our study. The subjects answered the following adapted question: “since beginning of this program, how would you describe the change (if any) in activity limitations and motor function related to your condition?” It was asked from 0 (i.e., worsened) and 1 (i.e., no change) to 6–7 (i.e. very much improved); 2–3 stands for minimally improved, and 4–5 stands for much improved.

### Data Analysis

SPSS 23 (IBM, New York, NY, USA) was used for statistical analysis. First, pair-wise comparisons were performed between the pre-baseline and baseline assessments with the Wilcoxon signed-rank test. Second, the outcome measures of the averaged pre-baseline and baseline time points were compared to post-assessment and follow-up assessment time points with the Wilcoxon signed-rank test. Third, a correction for multiple comparisons was calculated with the Bonferroni correction and a significance level of *P* ≤ 0.017 was set.

## Results

### Subject Characteristics and Motivational Factors

The clinical characteristics of the subjects with iSCI (mean age 60 ± SD 10.2 years) are presented in Table 1. Two of the subjects were AIS C and 10 AIS D. Because of medical issues unrelated to the study, subject P3 withdrew after agreeing to participate in the study. None of the subjects had any pain while playing the games or after the sessions.

The motivation after training was 8.6 ± 1.3. The most attractive games as voted by the patients were the following: Hamster Splash (number of subjects: 5), Star Kick and Planet Drive (2 each), and Footbag and Get to the Game (1 each). The most difficult and challenging game for all patients was “Planet Drive.” The subjects played mainly in a sitting position (chair or wheelchair) but changed to a standing position for the last repetitions of Footbag, Star Kick, and Get to the Game.

All subjects familiarized themselves with the VR system quite quickly. Overall, the subjects evaluated the system very positively. It was reported to be user-friendly and they liked the visual features. Some of the subjects could also imagine using the system for longer than the required 4 weeks and reported a positive effect such as being able to lift their feet better, improved sensation in the legs/feet, and improved swimming and walking.

### Outcomes—Training at Home (Home Based)

Between the time points pre-baseline and baseline, the outcome measures did not differ, which is an indication for outcome stability for the included subjects before the intervention. The values of the outcome measures are presented in Table 2 whereby one subject was unable to perform the walking assessments (P11).

At post-assessment, significant increases (*P* ≤ 0.017) in comparison with the averaged pre-baseline and baseline were found in the primary outcome measures such as muscle strength (LEMS, *P* = 0.008), balance (BBS, *P* = 0.008), and functional mobility (TUG, *P* = 0.005). In addition, 7 out of 11 subjects improved (i.e., an increase of one grade at least) in ankle dorsiflexion (L4) and four of them reached the MCID of LEMS (overall) at post-assessment. The secondary outcome measures showed with respect to walking speed/distance and mobility no significant effects: 10MWT (*P* = 0.169), 6minWT (*P* = 0.037); SCIM III mobility (*P* = 0.018), and WISCI II (*P* = 0.180). Concerning walking speed/distance, seven tested subjects met the limits for the MCID of 10MWT and one subject reached the MCID of 6minWT at post-assessment. In addition, a clinically important difference was shown in two subjects using WISCI II.

At follow-up assessment, a significant increase (*P* ≤ 0.017) in comparison with the averaged pre-baseline and baseline was found in functional mobility (TUG, *P* = 0.005). No significant changes were found in muscle strength (LEMS, *P* = 0.065) and balance (BBS, *P* = 0.28) as well as in walking speed/distance and mobility: 10MWT (*P* = 0.169), 6minWT (*P* = 0.32), SCIM III mobility (*P* = 0.026), and WISCI II (*P* = 0.317).

Eleven subjects rated their motor function using the adapted PGIC after the last day of treatment rated on a 7-point scale (Table 3). Seven subjects rated motor function as markedly improved (much improved or very much improved).

**Table 3:** Adapted Patients’ Global Impression of Change (PGIC).

PGIC	Number of subjects
Very much improved (6–7)	2
Much improved (4–5)	5
Minimally improved (2–3)	4
Worsened or no change (0–1)	0

## Discussion

The study assessed the feasibility and effectiveness of a home-based and first-person VR-augmented neurorehabilitation training for the lower limbs in subjects with chronic iSCI by combining action observation and execution. Overall, the training was well accepted by the patients and the results revealed that patients improved significantly in lower limb muscle strength, balance, and functional mobility.

### Motor Outcomes

Improvements were found in 64% of the subjects in ankle dorsiflexion (L4), and a general increase in lower limb muscle strength (LEMS) was achieved. In addition, an MCID by one-third of the subjects was reached. These changes may have been of relevance in enabling the observed gains in walking speed, stability, and mobility (reflected by improvements in TUG and BBS). However, it has been argued that changes in the LEMS, i.e., muscle strength, do not necessarily relate to changes in SCIM and WISCI (31). Reliance on walking aids and/or personal assistance evaluated by SCIM mobility and WISCI II seem not to be supported by the actual training. Along the same line, only two subjects showed clinically important differences using WISCI II and no significant overall effect.

Furthermore, longer walking distances included in the outcome measures SCIM mobility and 6minWT (only one subject reached MCID) might not be specifically trained by the performed VR tasks. Nevertheless, the TUG assesses various aspects of mobility which are correlated with performance in for example the 10MWT (one-third of the tested subjects met the limits for the MCID) in subjects with iSCI (23). In healthy elderly people, the TUG shows excellent correlation with the BBS and gait speed (20) and is moderately related to executive function (32). The BBS correlates well with other mobility measures and muscle strength (33, 34) and is important for postural control (35).

In accordance with the objective assessments, 64% of the subjects rated their motor functions as markedly improved (i.e., PGIC ≥ 4) when they were asked after the treatment: “since beginning of this program, how would you describe the change (if any) in activity limitations and motor function related to your condition?” The other tested subjects reported at least minimal improvements (i.e., PGIC 2 or 3). This is in opposition to a report claiming that evaluated functional improvements were not actually perceived as such by subjects with a SCI (36).

### Task Specificity, Dosage, and Motivation

Despite the task specificity of the training, i.e., isolated single-joint movements, subjects improved on global functional scores. In other words, this type of training with task- and deficit-specific variation enabled a transfer of gained function to other tasks (e.g., functional mobility). In line with our results, a study with lower limb strength training (12 weeks, 30 sessions) also showed a transfer of specific training to general improvements. They found attenuated neuromuscular impairments and improved locomotion in subjects with iSCI (37). Another study found that muscle strength training of the lower limbs generates improved results in walking-related outcome measures compared to robot-assisted gait training in subjects with iSCI with limited ambulatory function (38). Hence, these findings support our results that functional improvements, e.g., mobility, may also be feasible with no task-specific training.

The central idea of motor learning is repeating exercises, feedback about movement execution and motivation (12). When for example the ankle movements are calculated per leg per training session, the subjects executed around 300 movements each. This high number of repetitions, leading to increased internal stimulation of higher motor brain areas, might have induced plastic changes. It has been shown that improvements induced by motor training in healthy controls correlates with an increase in cortical volume in humans (39–43) and monkeys (44). The effects were stronger when a new task was learned and the changes occurred rather rapidly (within weeks) (39, 44). As in the current study, the action processing system is activated by visuo-motor tasks and its downstream motor areas are involved in movement execution, thus effectively promoting functional recovery (45). For subjects with chronic iSCI, there seems to be similar effects. Our group could recently show training-induced functional improvements (e.g., balance) accompanied by structural brain plasticity after intense VR-augmented training, i.e., isolated single-joint movements, for the lower limbs with a high number of repetitions (46). However, few clinical studies adequately address the issue of dosing and timing of lower and upper limb rehabilitation after SCI (47). Wirz et al. (10) demonstrated that longer trainings led to improved mobility in subjects with iSCI.

As mentioned earlier, beside the amount of training, active participation and motivation is a key element for achieving functional improvements (11). The motivation after training was quite high (8.6 on an 11-point NRS from 0 to 10), and no pain or spasticity was reported by the subjects playing the VR tasks. Playing games interactively with a high motivation and the combination of observation and motor imagery, i.e., being a part of the immersive environment, enhance the activation of sensorimotor networks (46, 48).

Interestingly, the clinic-based training, i.e., training at the hospital with one-to-one therapist–patient support, showed greater improvements of balance and walking capacity in the absolute assessment values as the currently tested home-based system (7). The setup in both studies was similar but differed in therapist presence during the training sessions. Therefore, human therapist presence and feedback may have an additional effect on subject’s motivation and should be further investigated.

### Study Limitations

The study described here is limited by the small and heterogeneous group of 12 subjects with iSCI (AIS C/D with various lesion levels), the uncontrolled study design and non-blinded assessment. While the pre-baseline did at least help to ensure that each subject was at a stable level before the beginning of the intervention, it is recommended to conduct a larger randomized controlled study to verify or refute the conclusions of this study.

## Conclusion and Implication

Virtual reality-based rehabilitation training combining action observation and execution and providing intensive, repetitive, and motivating training scenarios led to improvements in lower limb motor function. Therefore, the system may be of benefit as a neurorehabilitation tool. In addition, because the study design used unsupervised training at subjects’ homes, it suggests that the system can be successfully used in ecologically valid home-based training settings. Such a system may provide additional benefits in terms of reduced subject transportation cost and effort and for monitoring of subjects’ activity outside the clinic. This data may provide a window to gain insights into how the results of inpatient rehabilitation translate to post-clinic everyday life and track the maintenance, gain, or loss of function after the end of supervised rehabilitation.

The VR-augmented neurorehabilitation described in this study is not limited to subjects with iSCI. Other subject groups with neurological disorders, e.g., stroke, are also likely to benefit from the intervention described here. Furthermore, studies are needed to determine advantages, disadvantages, cost-effectiveness, and comparative efficacy of home-based versus clinic-based VR training.

## Ethics Statement

All authors declared no potential conflicts of interest with respect to the research, authorship, and/or publication of this article. The purpose of the study was clear to all subjects, and written informed consent was acquired. The experimental protocol was conducted in accordance with the Declaration of Helsinki and performed with the approval of the Canton of Zurich ethics committee (EK-24/2009, PB2016-00545).

## Author Contributions

MV was involved in study design, data collection, analysis of data, discussion of results, and writing the article. JL was involved in data collection, data analysis, and discussion of results. LA, RS, and RC were involved in the discussion of results and manuscript editing. PP and AC were involved in study design, discussion of results, and editing the article. KE and MB were involved in study design, data analysis, discussion of results, and article editing.

## Conflict of Interest Statement

All authors declared no potential conflicts of interest with respect to the research, authorship, and/or publication of this article. The experiment was conducted in accordance with the Declaration of Helsinki.
